# Reclassification of *Paenibacillus riograndensis* as a Genomovar of *Paenibacillus sonchi*: Genome-Based Metrics Improve Bacterial Taxonomic Classification

**DOI:** 10.3389/fmicb.2017.01849

**Published:** 2017-10-04

**Authors:** Fernando H. Sant’Anna, Adriana Ambrosini, Rocheli de Souza, Gabriela de Carvalho Fernandes, Evelise Bach, Eduardo Balsanelli, Valter Baura, Luciana F. Brito, Volker F. Wendisch, Fábio de Oliveira Pedrosa, Emanuel M. de Souza, Luciane M. P. Passaglia

**Affiliations:** ^1^Departamento de Genética, Instituto de Biociências, Universidade Federal do Rio Grande do Sul, Porto Alegre, Brazil; ^2^Departamento de Bioquímica e Biologia Molecular, Centro Politécnico, Universidade Federal do Paraná, Curitiba, Brazil; ^3^Department of Genetics of Prokaryotes, Faculty of Biology & CeBiTec, Bielefeld University, Bielefeld, Germany

**Keywords:** *Paenibacillus*, *Paenibacillus riograndensis*, *Paenibacillus sonchi*, taxonomy, average nucleotide identity, dDDH, phylogeny

## Abstract

Species from the genus *Paenibacillus* are widely studied due to their biotechnological relevance. Dozens of novel species descriptions of this genus were published in the last couple of years, but few utilized genomic data as classification criteria. Here, we demonstrate the importance of using genome-based metrics and phylogenetic analyses to identify and classify *Paenibacillus* strains. For this purpose, *Paenibacillus riograndensis* SBR5^T^, *Paenibacillus sonchi* X19-5^T^, and their close relatives were compared through phenotypic, genotypic, and genomic approaches. With respect to *P. sonchi* X19-5^T^, *P. riograndensis* SBR5^T^, *Paenibacillus* sp. CAR114, and *Paenibacillus* sp. CAS34 presented ANI (average nucleotide identity) values ranging from 95.61 to 96.32%, gANI (whole-genome average nucleotide identity) values ranging from 96.78 to 97.31%, and dDDH (digital DNA–DNA hybridization) values ranging from 68.2 to 73.2%. Phylogenetic analyses of 16S rRNA, *gyrB, recA, recN*, and *rpoB* genes and concatenated proteins supported the monophyletic origin of these *Paenibacillus* strains. Therefore, we propose to assign *Paenibacillus* sp. CAR114 and *Paenibacillus* sp. CAS34 to *P. sonchi* species, and reclassify *P. riograndensis* SBR5^T^ as a later heterotypic synonym of *P. sonchi* (type strain X19-5^T^), with the creation of three novel genomovars, *P. sonchi* genomovar Sonchi (type strain X19-5^T^), *P. sonchi* genomovar Riograndensis (type strain SBR5^T^), *P. sonchi* genomovar Oryzarum (type strain CAS34^T^ = DSM 102041^T^; = BR10511^T^).

## Introduction

The genus *Paenibacillus* includes nitrogen-fixing species that were isolated from roots of wheat (Beneduzi et al., 2010), maize (Berge et al., 2002), rice (Beneduzi et al., 2008; de Souza et al., 2014), and other plants (Ambrosini et al., 2015). Many of these bacterial isolates arose from the search for strains that exert positive effects on the development of agricultural plants. The inoculation of plants or seeds with these plant-growth promoting bacteria (PGPB) can help to reduce the use of chemical fertilizers and pesticides, improving agricultural sustainability (Beneduzi et al., 2008; Seldin, 2011).

In 2010, the species *Paenibacillus riograndensis* (Beneduzi et al., 2010) and *Paenibacillus sonchi* (Hong et al., 2009) were described in an interval of only 9 days, according to electronic publication dates. *P. riograndensis* is a nitrogen fixer and phytormone producer (Beneduzi et al., 2010), which in later studies was found to be very closely related to *P. sonchi*, although their metabolic repertoire seemed to be distinct (Jin et al., 2011). Recently, the complete genome sequence of *P. riograndensis* SBR5^T^ (Brito et al., 2015) and a draft genome sequence of *P. sonchi* X19-5^T^ (Xie et al., 2014) were determined, and preliminary computation of their ANI (average nucleotide identity) suggested that they belong to the same species.

Genomic data are portable (i.e., data obtained from different laboratories can be compared), and highly informative with respect to evolutionary relationships, which are desired features for any taxonomic scheme. Although high-throughput sequencing technologies facilitated access to genome sequences, taxonomic reports rarely present this kind of data. For example, since 2016, from more than 20 novel *Paenibacillus* species descriptions in the International Journal of Systematic and Evolutionary Microbiology, only one of them utilized genomic analyses (Liu et al., 2016).

Here, we demonstrate the importance of using genomic analyses to clarify the taxonomic assignment of *P. riograndensis* and *P. sonchi*. For this purpose, we compared *P. riograndensis* SBR5^T^, *P. sonchi* X19-5^T^, and their close relatives, *Paenibacillus graminis* DSM 15220^T^, *Paenibacillus jilunlii* DSM 23019^T^, and two strains preliminarily identified as *P. riograndensis/P. sonchi* (*Paenibacillus* sp. CAS34 and *Paenibacillus* sp. CAR114) through genomic, genotypic, and phenotypic approaches.

## Materials and Methods

### Bacterial Strains

*Paenibacillus graminis* DSM 15220^T^ (= RSA19^T^) and *Paenibacillus jilunlii* DSM 23019^T^ were purchased from Deutsche Sammlung von Mikroorganismen und Zellkulturen (DSMZ), Braunschweig, Germany. *P. sonchi* X19-5^T^ (= LMG 24727^T^) was acquired from Belgian Coordinated Collections of Microorganisms – Laboratory for Microbiology of Ghent University (BCCM/LMG), Ghent, Belgium. *P. riograndensis* SBR5^T^ (= CCGB 1313^T;^ = CECT 7330^T^) (Beneduzi et al., 2010), *Paenibacillus* sp. CAR114 (= DSM 102250; = BR10512), and *Paenibacillus* sp. CAS34 (= DSM 102041; = BR10511) were obtained from our bacterial collection. *Paenibacillus* sp. CAR114 and CAS34 strains were originally isolated from roots of rice (*Oryza sativa*) cultivated in Cachoeirinha, Southern Brazil (de Souza et al., 2014). *Paenibacillus polymyxa* ATCC 842^T^ (= BGSC 25A1^T^), the type strain of the genus *Paenibacillus*, was purchased from Bacillus Genetic Stock Center (BGSC).

### Culture Conditions and Biochemical Tests

All *Paenibacillus* strains were routinely grown in King B broth [Peptone 20 g/liter; K_2_HPO_4_ 1.15 g/liter; MgSO_4_.7H_2_O, 1.5 g/liter; Glycerol, 1.5% (vol/vol)] at 28°C previously to the physiological tests (Glickmann and Dessaux, 1995). Morphological and biochemical characterization were carried out as described in standard protocols (Holt, 1986, 2000; MacFaddin, 2000). For biochemical profiling, five independent experiments were performed. As described elsewhere, bacterial strains were also tested for the production of indolic compounds (IC) (Glickmann and Dessaux, 1995), siderophores (Schwyn and Neilands, 1987), and for the ability to perform biological nitrogen fixation (Boddey and Knowles, 2008; Ambrosini et al., 2012). Three independent cultures were evaluated for each assay.

### Multivariate Analyses Based on Phenotypic Data

Principal component analysis (PCA) was used to verify the statistical variance-covariance of cellular fatty acids among five *Paenibacillus* species through PAST software (Hammer et al., 2001).

### Genome Sequences

All genomes utilized in this study are listed in Supplementary Table S1. Genome sequences of *P. jilunlii* DSM 23019^T^, *Paenibacillus* sp. CAR114 and *Paenibacillus* sp. CAS34 were obtained using the Illumina Miseq platform. Total DNA was extracted as described elsewhere (Ambrosini et al., 2012). Libraries were constructed using Nextera XT kit and sequenced with MiSeq Reagent Kit V3 (2 × 300 bp). Draft genomes were assembled using SPAdes version 3.5 (careful option and k-mer values equal to 21, 33, 55, 77, 99, 127) (Bankevich et al., 2012), and annotated using the NCBI Prokaryotic Genome Automatic Annotation Pipeline (PGAAP), which combines Hidden Markov Model (HMM)-based gene prediction methods with homology-based methods (Angiuoli et al., 2008).

Genome assembly quality was assessed with Quast version 2.3 (Gurevich et al., 2013) and Checkm version 0.9.6 (Parks et al., 2014).

R2cat (Husemann and Stoye, 2010) is a contig arrangement tool used to order a set of contigs with respect to a single reference genome, in which mapping of the contigs onto the reference is based on a q-gram filter. The results are visualized by plotting contigs onto the reference sequence of a complete genome. EDGAR (Efficient Database framework for comparative Genome Analyses using BLAST score Ratios) was utilized to automatically perform genome comparisons in a high throughput approach (Blom et al., 2009). EDGAR generates synteny plots, describing the physical co-localization of genes, which may change order during evolution by rearrangement events like inversions, deletions, insertions, or translocations.

Protein counterparts of *Paenibacillus* strains were detected in *P. riograndensis* SBR5 translated genome sequence through tblastn searches, implemented in BRIG software version 0.95 (Alikhan et al., 2011).

### 16S rRNA, *gyrB, recA, recN*, and *rpoB* Gene Phylogenies

16S rRNA gene sequences of *Paenibacillus* type-strains were retrieved from RDP (Ribosomal Database Project) (Cole et al., 2014). 16S rRNA genes were also obtained from annotated genomes (Supplementary Table S1). 16S rRNA gene sequences were aligned using SINA software version 1.2.11 with default parameters (Pruesse et al., 2012). The 16S rRNA gene alignment without gaps contained 775 positions. The *gyrB, recA, recN*, and *rpoB* genes were retrieved from the genomes (Supplementary Table S2), and aligned using MUSCLE software version 3.8.31 using default parameters (Edgar, 2004). Positions containing gaps of the sequence alignments were removed. The *gyrB, recA, recN*, and *rpoB* alignments without gaps contained 1911, 1059, 1215 and 2596 positions, respectively.

Maximum-likelihood phylogenies were conducted with the Phylogeny.fr platform with the PhyML program (v3.0 aLRT) (Dereeper et al., 2008). GTR (Generalized Time Reversible) substitution model was selected assuming an estimated proportion of invariant sites and four gamma-distributed rate categories to account for rate heterogeneity across sites. Gamma shape parameters were estimated directly from the data. Reliability for internal branching was assessed using the aLRT (approximate Likelihood Ratio Test) (Anisimova and Gascuel, 2006). All phylogenetic trees were rooted using *P. polymyxa* ATCC 842^T^ as outgroup. The 16S rRNA gene phylogenetic tree was pruned using Newick Utilities (Junier and Zdobnov, 2010), maintaining strains closely related to the *P. riograndensis* and *P. sonchi* species.

### Core-Proteome and AMPHORA Multiprotein Phylogenetic Reconstructions

Ortholog protein groups were defined using bidirectional best hits algorithm implemented in Get_homologues build 20170609. For this purpose, the core-proteome was compiled using minimum BLAST searches and clusters containing inparalogs were excluded. Each of the 1102 single-copy proteins was aligned with MUSCLE software using default parameters. Alignments were concatenated with Phyutility (Smith and Dunn, 2008). Phylogenetic tree of the core-proteome was reconstructed with Mega 6 software build 6140226 (Tamura et al., 2013), using Neighbor Joining approach with Jones-Taylor-Thornton substitution model, deleting positions containing gaps and using 500 bootstrap replicates.

Thirty-one marker proteins were detected in the genomes using Amphora pipeline (Wu and Eisen, 2008) implemented at the AmphoraNet (Kerepesi et al., 2014). Since our analysis included draft genome sequences, few protein sequences were found fragmented in more than one contig. Therefore, if more than one hit for a protein was found in a genome, the largest one was kept for subsequent analyses in order to maximize the information for phylogenetic inference. All Amphora proteins are listed in Supplementary Table S3. Proteins were aligned and concatenated as described above. Aligned positions were curated using Gblocks (Castresana, 2000) with default parameters (implemented in Phylogeny.fr pipeline). The concatenated alignment without gaps containing 6289 positions. Phylogenetic analysis of AMPHORA concatenated multiprotein sequence was performed with the Phylogeny.fr platform as described in the previous section, although the WAG substitution model was utilized in this case.

Both phylogenies were rooted using *P. polymyxa* ATCC 842^T^ as the outgroup.

### ANI, dDDH, and MiSI Estimations

Average nucleotide identity values based on Blast alignments from all pairwise genome comparisons were computed at JspeciesWS^1^ (Richter and Rosselló-Móra, 2009). dDDH values were estimated at GGDC (Genome-to-Genome Distance Calculator)^2^ using GGDC 2.0 BLAST+ and recommended formula 2 (Meier-Kolthoff et al., 2013a). MiSI method was utilized as described in Varghese et al. (2015). For this purpose, all CDS from each genome were extracted using a script written in BioPython (Cock et al., 2009), which can be downloaded from https://github.com/fhsantanna/bioinfo_scripts.

## Results

### Phenotypic Analyses

The type strains of *P. graminis* and *P. jilunlii*, and two strains, *Paenibacillus* sp. CAR114 and *Paenibacillus* sp. CAS34, which are all closely related to *P. riograndensis* and *P. sonchi*, had their phenotypic characteristics evaluated. **Table 1** and Supplementary Table S4 show the biochemical capabilities of these bacteria. Concerning the tests performed, *P. sonchi* X19-5^T^ and *P. riograndensis* SBR5^T^ only diverged with respect to starch degradation.

**Table 1:** Comparison of biochemical characteristics of *Paenibacillus* strains.

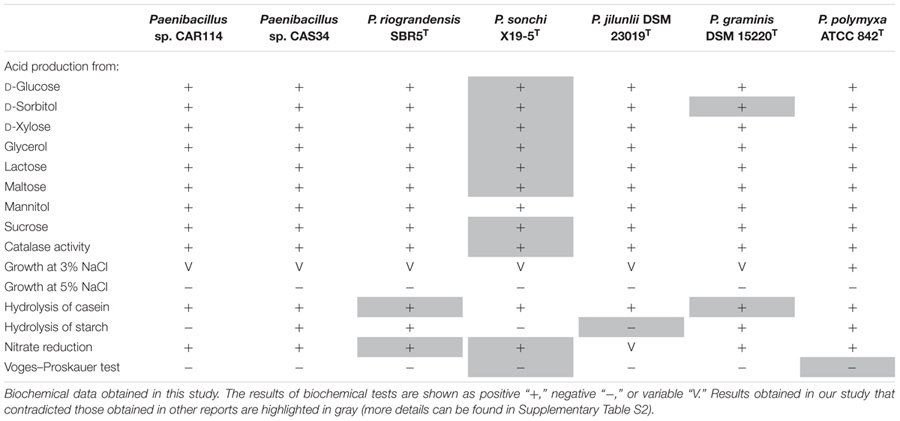

As demonstrated in Supplementary Table S5, *Paenibacillus* sp. CAR114 and *Paenibacillus* sp. CAS34 were able to fix nitrogen and synthesize indolic compounds as *P. sonchi* X19-5^T^ and *P. riograndensis* SBR5^T^.

Considering each species at a time in the scatter plot generated from multivariate analysis of the fatty acid profiles, the points representing independent profiles of *P. graminis* DSM 15220^T^, *P. jilunlii* DSM 23019^T^ and *P. sonchi* X19-5^T^ were more distant from each other than those from the other *Paenibacillu*s strains (Supplementary Figure S1 and Table S6).

### 16S rRNA Gene Analyses

**FIGURE 1:**
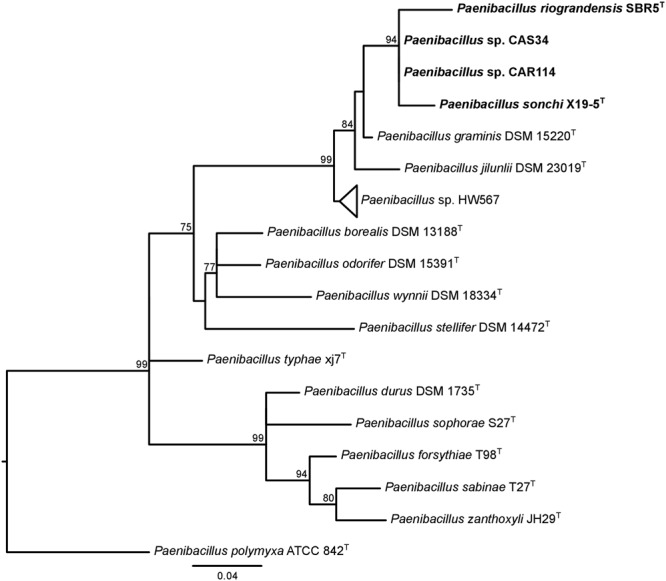
16S rRNA gene phylogeny of *Paenibacillus* species. The 16S rRNA gene rooted tree was constructed using the maximum-likelihood method. aLRT values greater than 70% are shown next to the branches. The tree is drawn to scale, with branch lengths in the same units as those of the evolutionary distances used to infer the phylogenetic tree. *P. polymyxa* is the outgroup. Bacteria of *P. riograndensis/P. sonchi* clade are in bold. This tree is the same of Supplementary Figure S2, although only taxa of interest were kept.

The 16S rRNA genes were analyzed concerning their identity levels and phylogenetic history. *P. sonchi, P. riograndensis, P. graminis*, and *P. jilunlii* strains shared 16S rRNA gene identity values higher or equal than the species cutoff (Supplementary Table S7). In the 16S rRNA gene phylogeny containing all sequences from *Paenibacillus* type-species deposited in RDP, *P. sonchi* X19-5^T^, *P. riograndensis* SBR5^T^, *Paenibacillus* sp. CAR114 and *Paenibacillus* sp. CAS34 formed a clade (**Figure 1** and Supplementary Figure S2). However, no subclades were present in this monophyletic group. It is worth noting that both *P. riograndensis* SBR5^T^ and *P. graminis* DSM 15220^T^ have multiple copies of 16S rRNA gene (9 and 10, respectively) in their complete sequenced genomes (not all copies were available from strains with draft genomes). Even though 16S rRNA gene paralogs were distinct, they were not variable enough to be dispersed in different clades (Supplementary Figure S2).

### Genomic Analyses

For some genomic analyses, we also considered other *Paenibacillus* strains from sister groups of *P. riograndensis-P. sonchi-P. graminis-P. jilunlii* cluster, whose genome sequences were publicly available (Supplementary Table S1).

**FIGURE 2:**
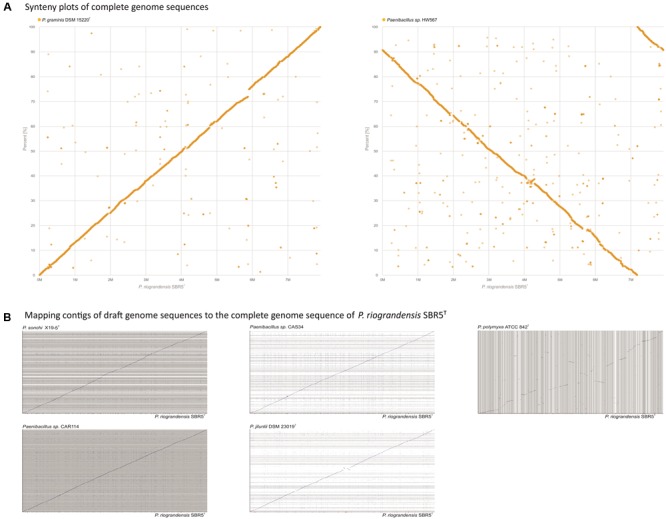
Synteny analysis of *P. riograndensis* SBR5^T^ and the complete genome sequences of related species **(A)**, and mapping of draft genome sequences of related species to the *P. riograndensis* SBR5^T^ genome **(B)**. Blue line denotes syntenic scaffold alignments. The horizontal bar at the bottom indicates coverage of the matches to the reference scaffold, SBR5^T^, with maximal coverage indicated with black, fading to light gray with less coverage. Red indicates uncovered regions.

The G+C content of *Paenibacillus* strains ranged from 44.2 to 53.5%, although the values among *P. graminis* DSM 15220^T^, *P. jilunlii* DSM 23019^T^, *P. riograndensis* SBR5^T^, *P. sonchi* X19-5^T^, *Paenibacillus* sp. CAR114, and *Paenibacillus* sp. CAS34 did not vary more than 1 percentual point (Supplementary Table S1). In order to analyze genome structural conservation in selected *Paenibacillus* species, we generated synteny plots to compare the complete genome sequence of *P. riograndensis* SBR5^T^ to those from *P. graminis* DSM 15220^T^ and *Paenibacillus* sp. HW567 (**Figure 2A**). Also, the draft genome sequences of *P. sonchi* X19-5^T^, *Paenibacillus* sp. CAR114, *Paenibacillus* sp. CAS34, *P. graminis* DSM 15220^T^, *P. jilunlii* DSM 23019^T^, and *P. polymyxa* ATCC 842^T^ were mapped to the complete genome sequence of *P. riograndensis* SBR5^T^ (**Figure 2B**). With the exception of *P. polymyxa* ATCC 842^T^ graph, the plots presented long diagonal lines, interrupted only by few short gaps (**Figures 2A,B**).

**FIGURE 3:**
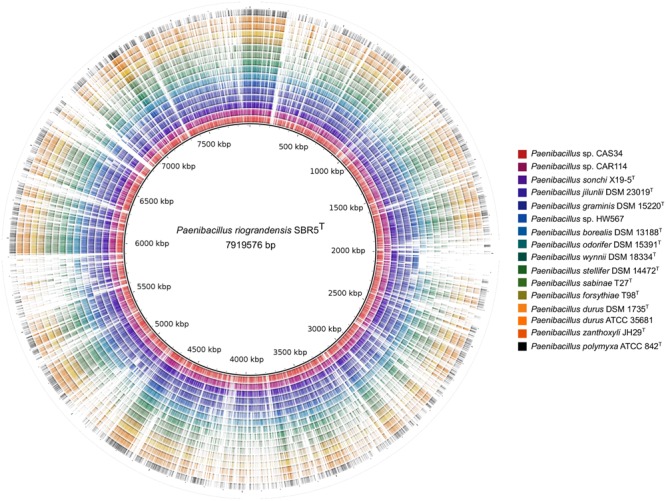
Comparison of predicted proteins of *Paenibacillus* spp. against the *P. riograndensis* SBR5 translated genome sequence. The innermost black line circle represents the *P. riograndensis* SBR5 genome sequence. Each one of the outer rings represents a *Paenibacillus* strain, as depicted in the legend box. Colored regions of each ring symbolize tblastn hits with at least 30% of identity.

The content of homolog proteins of the *Paenibacillus* strains in relation to the translated genome sequence of *P. riograndensis* SBR5^T^ was also verified (**Figure 3**). Closely related strains, represented by the six inner rings in **Figure 3**, presented more color dense regions than other strains. On the other hand, the 10 outer rings of **Figure 3**, representing relatively more distant strains in relation to *P. riograndensis* SBR5^T^, presented more gaps.

Genomic metrics computed from comparisons among *P. riograndensis* SBR5^T^, *P. sonchi* X19-5^T^, *Paenibacillus* sp. CAR114, and *Paenibacillus* sp. CAS34 presented values above the species circumscription thresholds (**Table 2** and Supplementary Tables S8–S10).

**Table 2:** Comparison of *P. sonchi* X19-5^T^ with *Paenibacillus* strains through different whole-genome based methods.

	ANI [AN]	MiSI (gANI [AF])	dDDH [CI]
*P. riograndensis* SBR5^T^	**96.28 [77.67]**	**97.31 [0.78]**	**73.2 [±2.92]**
*Paenibacillus* sp. CAR114	**95.61 [70.63]**	**96.78 [0.6]**	68.2 [**±**2.92]
*Paenibacillus* sp. CAS34	**96.32 [78.48]**	**97.29 [0.76]**	**72.8 [±2.92]**
*P. graminis* DSM 15220^T^	91.70 [72.48]	93.34 [0.69]	51.6 [**±**2.66]
*P. jilunlii* ATCC 23019^T^	92.99 [75.94]	94.33 [0.75]	53.1 [**±**2.69]
*P. polymyxa* ATCC 842^T^	68.84 [24.94]	72.1 [0.22]	22.1 [**±**2.36]
*Paenibacillus* sp. HW567	81.01 [54.58]	83.09 [0.55]	26.9 [**±**2.42]

*AN, aligned nucleotides; AF, alignment fraction; CI, confidence interval.*

*Values in bold are higher than the threshold for species demarcation: ANI ≥ 95%, gANI ≥ 96.5%, and dDDH ≥ 70%.*

Although the dDDH value of *Paenibacillus* sp. CAR114 versus *P. sonchi* X19-5^T^ was 68.2%, the superior limit of the confidence interval (71.12%) surpassed the dDDH species threshold of 70% (**Table 2**). *Paenibacillus* sp. CAR114 and *Paenibacillus* sp. CAS34 presented dDDH values higher than the subspecies circumscription threshold of 79% between themselves, but lower than that in relation to *P. riograndensis* SBR5^T^ and *P. sonchi* X19-5^T^ (Supplementary Table S10). Furthermore, *P. riograndensis* SBR5^T^ and *P. sonchi* X19-5^T^ presented 73.2% of dDDH, below the subspecies threshold (**Table 2**).

**FIGURE 4:**
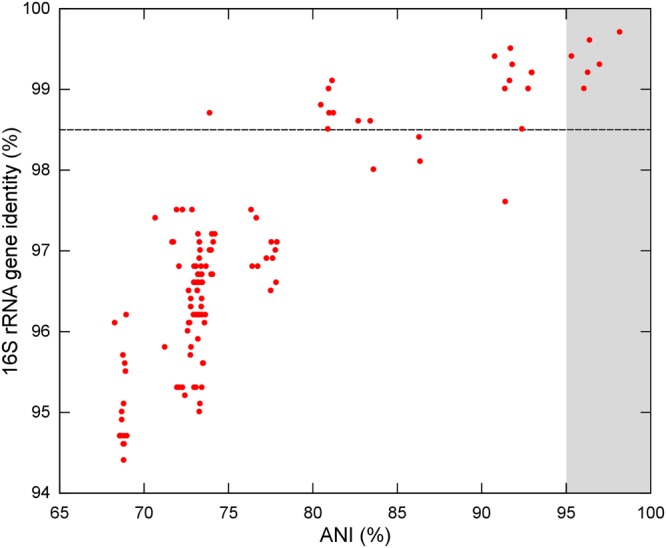
Relationship between ANI and 16S rRNA gene identity levels among *Paenibacillus* species. Each red circle represents a comparison between two *Paenibacillus* species. Traced line shows the 16S rRNA gene identity threshold for species demarcation. Gray background represents the ANI range for species demarcation. All six red circles over the gray background are pairwise comparisons of *P. riograndensis, P. sonchi, Paenibacillus* sp. CAR114, and *Paenibacillus* CAS34.

**Figure 4** and Supplementary Table S11 show the correspondence between the 16S rRNA gene identity values and ANI values computed in a pairwise manner. Dozens of strain-strain comparisons presented 16S rRNA gene identity values higher than the species threshold for this marker, although their ANI values were below 95%. Only those comparisons among *P. riograndensis* SBR5^T^, *P. sonchi* X19-5^T^, *Paenibacillus* sp. CAR114 and *Paenibacillus* CAS34 presented values above the ANI and 16S rRNA gene identity thresholds.

### Phylogenetic Analyses

**FIGURE 5:**
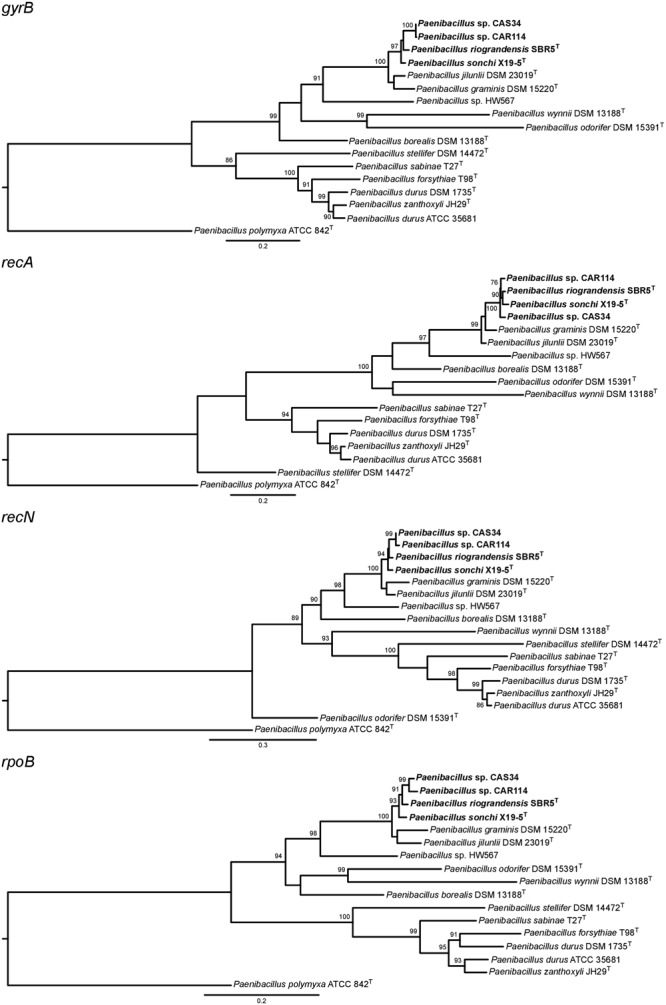
Phylogenies of genes other than 16S rRNA gene of *Paenibacillus* species. The trees were built using the maximum-likelihood method. Details are as shown in **Figure 1**, unless specified otherwise.

**FIGURE 6:**
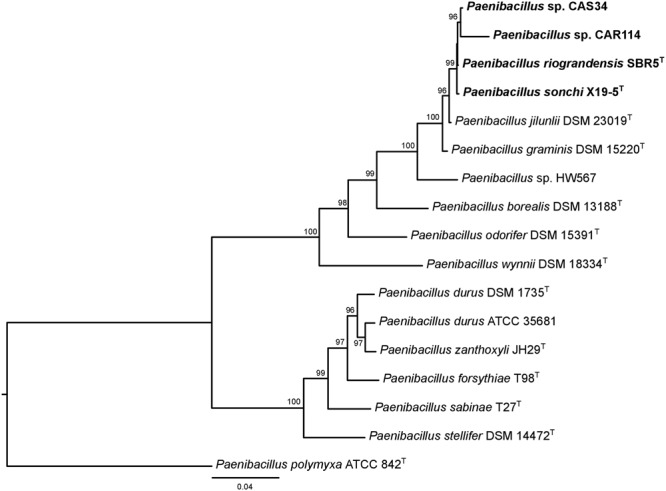
AMPHORA multiprotein phylogeny of *Paenibacillus* species. The multiprotein rooted tree was constructed using the maximum-likelihood method. Details are as shown in **Figure 1**, unless specified otherwise.

**FIGURE 7:**
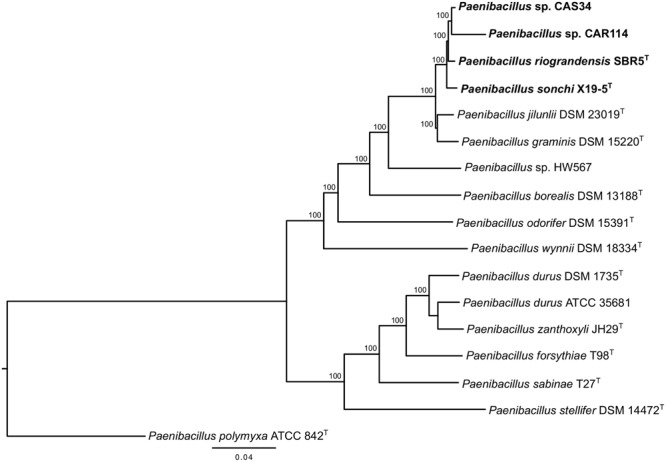
Core-proteome phylogeny of *Paenibacillus* species. The core-proteome rooted tree was constructed using the Neighbor Joining method. Details are as shown in **Figure 1**, unless specified otherwise. Bootstrap values greater than 70% are shown next to the branches.

The phylogenetic history of *P. sonchi, P. riograndensis*, and their closely related species was also reconstructed utilizing *gyrB, recA, recN* and *rpoB* genes, a combined dataset of 31 marker proteins (AMPHORA pipeline) and the concatenated core-proteome. The clade composed of *P. riograndensis* SBR5^T^, *P. sonchi* X19-5^T^, *Paenibacillus* sp. CAR114 and *Paenibacillus* CAS34 was consistent in all of these phylogenetic reconstructions (**Figures 5–7**), as well as in the 16S rRNA gene phylogeny (**Figure 1**). Furthermore, with the exception of *recA* phylogeny, all trees contained a subclade of *Paenibacillus* sp. CAR114 and *Paenibacillus* sp. CAS34, which grouped with *P. riograndensis* SBR5^T^. However, this latter grouping pattern was only supported in the *rpoB* gene and core-proteome phylogenies.

## Discussion

Polyphasic taxonomy relies on phenotypic and genotypic analyses to identify and classify bacterial specimens. In line with this principle, we investigated different characteristics of *P. riograndensis* and *P. sonchi* in order to clarify their taxonomic statuses. For this purpose, we performed comparative analyses using closely related species to *P. riograndensis* and *P. sonchi* as references. As observed by Sutcliffe et al. (2012), most publications describing new prokaryotic taxa are based on only one representative strain. To overcome this limitation, we included in our analyses the strains *Paenibacillus* sp. CAR114 and *Paenibacillus* sp. CAS34, preliminarily identified as *P. sonchi/P. riograndensis* based on 16S rRNA gene comparisons. Both strains were isolated from rice rhizosphere (de Souza et al., 2014), and were able to fix nitrogen and synthesize indolic compounds as *P. sonchi* X19-5^T^ and *P. riograndensis* SBR5^T^. These features are commonly implicated with plant-growth promotion (Lugtenberg and Kamilova, 2009).

For biochemical profiling, we selected tests in which *P. sonchi* X19-5^T^ and *P. riograndensis* SBR5^T^ were initially distinguishable, based on their species description reports. However, in our experiments not only *P. sonchi* X19-5^T^ and *P. riograndensis* SBR5^T^ presented similar biochemical profiles, but also all strains evaluated did (variation among strains occurred in few tests). In fact, our results are in contrast to some previously published biochemical profiles of *Paenibacillus* species. For instance, from all *Paenibacillus* strains considered here, only *P. sonchi* X19-5^T^ would be unable to produce acid from D-glucose, D-xylose, glycerol, lactose, and maltose. Nevertheless, despite the original description of *P. sonchi* (Hong et al., 2009), we verified through five independent experiments that its strain X19-5^T^ is actually able to form acid from these carbon sources. Another finding was that *P. riograndensis* SBR5^T^ is indeed capable to reduce nitrate, as predicted by genome sequence analysis (Brito et al., 2015), contradicting its original report (Beneduzi et al., 2010). As a matter of fact, divergent biochemical profiles were relatively common among different published reports, and at least one of them was problematic because of typographic errors. Therefore, taking these observations into account, at least for the *Paenibacillus* species studied here, biochemical profiling lacked reproducibility.

This same problem was found while revising fatty acid profiles from independent taxonomic reports, which presented many inconsistencies among *Paenibacillus* strains. Although fatty acid profiling is an essential prerequisite for bacterial species description, at least in our revision it did not prove to be portable, a desirable feature for chemotaxonomic characters (Logan et al., 2009).

Bacterial identification based on phenotypic traits tend to be less accurate than identification based on genotypic methods (Maughan and Van der Auwera, 2011). First, species may be composed of phenotypically heterogeneous strains (Kumar et al., 2015), i.e., sometimes it is difficult to obtain a common phenotypic pattern among strains of a species. Besides that, reliable phenotypic data are only obtained when strains are assayed simultaneously under carefully controlled culture conditions (Rosselló-Móra, 2012), since minimum variations can affect gene expression, and consequently, the phenotype. Moreover, as such data are usually descriptive, their criteria for species circumscription are not clear; therefore they should be carefully inspected for taxonomic classification purposes.

The homogeneity of *P. riograndensis, P. sonchi, P. graminis*, and *P. jilunlii* was also observed regarding their genotypic and genomic traits. Based on the correlation between 16S rRNA identity and genomic relatedness, it was recommended that a 16S rRNA gene identity of ∼98.5% is the adequate minimum threshold for species demarcation (Stackebrandt and Ebers, 2006; Meier-Kolthoff et al., 2013b; Kim et al., 2014). However, this recommendation was not applicable to differentiate the species *P. graminis, P. jilunlii, P. sonchi*, and *P. riograndensis*, whose 16S rRNA genes are highly conserved. As demonstrated by other studies, depending on the organisms investigated, 16S rRNA gene may not be a proper taxonomic marker to discriminate organisms at the species level (Fox et al., 1992; Chan et al., 2012).

Similarly, G+C content is also a characteristic with limited taxonomic value for these *Paenibacillus* species. A G+C content variation of at most 1 percentual point is expected for intra-species comparisons computed from genome sequences (Meier-Kolthoff et al., 2014b). However, the G+C contents of *P. graminis* DSM 15220^T^, *P. jilunlii* DSM 23019^T^, *P. riograndensis* SBR5^T^, *P. sonchi* X19-5^T^, *Paenibacillus* sp. CAR114, and *Paenibacillus* sp. CAS34 are very similar, not varying more than 1 percentual point among themselves. It is worth noting that the G+C content of *P. riograndensis* SBR5^T^ and *P. sonchi* X19-5^T^ were originally measured as 55.1 and 46.8%, respectively. This difference is substantial, since at least 10 percentual points of difference is expected to be found in species of distinct genus (Schleifer, 2009). However, nucleotide compositions were determined using indirect methods, which are less accurate in relation to those obtained using genomic data (Meier-Kolthoff et al., 2014b).

The structural conservation over the whole length of genomes from *P. riograndensis* SBR5^T^ and other *Paenibacillus* strains, namely *P. sonchi* X19-5^T^, *P. jilunlii* DSM 23019^T^, *P. graminis* DSM 15220^T^, *Paenibacillus* sp. CAR114, and *Paenibacillus* sp. CAS34 was discernible, and the genomic collinearity and proteomic conservation denoted the close phylogenetic relationship among these strains. Indeed, these *Paenibacillus* strains were only discriminated using methods based on genomic metrics. In this sense, whole genome data of *P. riograndensis* SBR5^T^, *P. sonchi* X19-5^T^, *Paenibacillus* sp. CAR114, and *Paenibacillus* sp. CAS34 generated by ANI, MiSI and dDDH were congruent. Therefore, these four bacterial strains would compose a single species, *P. sonchi*, which has name priority, since it was published first. Moreover, the results confirmed that *P. graminis* DSM 15220^T^ and *P. jilunlii* DSM 23019^T^ belong to other species.

Genome based metrics showed better resolution than 16S rRNA gene identity values. In fact, the resolving power of genome based methods was already explored to discriminate taxa at the infra-specific level. Meier-Kolthoff et al. (2014a) suggested that bacterial subspecies could be discerned based on dDDH values higher than 79%. Given this, *P. sonchi* could be divided in three subspecies, one harboring *P. sonchi* X19-5^T^, other harboring *P. riograndensis* SBR5^T^, and finally, another one containing *Paenibacillus* sp. CAR114 and *Paenibacillus* sp. CAS34. Although a quantitative measure is an important initiative for subspecies definition, bacterial taxonomy still relies on the interpretation of phenotypic data for this purpose. Therefore, these strains could not be considered subspecies, but genomovars, i.e., they represent genotypic entities at the subspecies level, but they do not present differential phenotypic characters required for categorization as subspecies (Schloter et al., 2000).

Gevers et al. (2005) stated that threshold methods such as ANI and dDDH could fail for delineating species when they contradict species phylogeny (for example, species having ANI values greater than 95% may not form a monophyletic group). Therefore, for taxonomic purposes, it is indispensable for ANI and dDDH values to be assisted by phylogenetic analyses.

The 16S rRNA gene phylogeny supported the results found in analyses based on genomic metrics, since *P. riograndensis* SBR5^T^, *P. sonchi* X19-5^T^, *Paenibacillus* sp. CAR114 and *Paenibacillus* sp. CAS34 formed a clade. This phylogenetic reconstruction showed that *P. sonchi* and *P. riograndensis* are closer to each other than to any other *Paenibacillus* species.

Logan et al. (2009) suggested that genes other than 16S rRNA gene could provide higher resolution for taxonomic analyses at species level. Indeed, the evolutionary relationships among *P. riograndensis* SBR5^T^, *P. sonchi* X19-5^T^, *Paenibacillus* sp. CAR114 and *Paenibacillus* sp. CAS34 were not clear in 16S rRNA gene phylogeny. Phylogenetic reconstructions based on *gyrB, recA, recN* and *rpoB* genes, and concatenated multiprotein sequences confirmed the monophyly of *P. riograndensis* SBR5^T^, *P. sonchi* X19-5^T^, *Paenibacillus* sp. CAR114, and *Paenibacillus* sp. CAS34. Furthermore, they also were more discriminative than the 16S rRNA gene phylogeny. With the exception of *recA* phylogeny, the closest relationship of *Paenibacillus* sp. CAR114 and *Paenibacillus* sp. CAS34 predicted by genome-genome comparisons was also demonstrated in these phylogenetic reconstructions.

All above findings strongly support that *Paenibacillus* sp. CAR114 and *Paenibacillus* sp. CAS34 belong to *P. sonchi* species, and that *P. riograndensis* is a later heterotypic synonym of *P. sonchi*. Considering phylogenetic reconstructions and dDDH values, we propose that three *P. sonchi* genomovars should be defined. Since genome approaches were essential for taxonomic classification of these *Paenibacillus* species, we consider that genomic metrics and phylogenies of genes other than 16S rRNA gene should be compulsory in the guidelines for describing new taxa.

### Emended Description of *Paenibacillus sonchi* Hong et al. (2009)

*Paenibacillus sonchi* (son’chi. L. n. sonchus -i, the herb sow-thistle, and also a botanical genus name; L. gen. n. sonchi of *Sonchus*, referring to the plant *Sonchus oleraceus*, the source of the rhizosphere soil from which the type strain was isolated).

Description as that given by Hong et al. (2009), except for the following modifications. Acid is produced from glucose, sucrose, lactose, D-xylose, maltose, D-sorbitol. Catalase-positive. Nitrate is reduced to nitrite. The G+C content of the DNA of the type strain X19-5^T^ is 50.36 mol%.

## Data Accessibility

The Whole Genome Shotgun projects have been deposited at DDBJ/ENA/GenBank under the accession numbers: LIRA00000000, for *P. riograndensis* CAR114, LIRB00000000 for *P. riograndensis* CAS34 and LIPY00000000 for *P. jilunlii* DSM 23019^T^. Phylogenetic data were deposited at TreeBASE under the accession URL http://purl.org/phylo/treebase/phylows/study/TB2:S18337.

## Author Contributions

FS and LP conceived and designed the experiments. FS, AA, LB, VW, and EdS wrote the paper. RdS, GdC, and EvB performed the biochemical assays. EdB and VB carried out the genome sequencing. EdS and FdO contributed with reagents and equipments. LB and VW performed the synteny analysis. AA performed the PCA of fatty acid profiles and analyzed the biochemical profiles of *Paenibacillus* strains. FS performed most of comparative genome and phylogenetic analyses and generated most of the artwork and tables. FS, AA, EvB, VW, EdS, and LP discussed the data and reviewed the manuscript.

## Conflict of Interest Statement

The authors declare that the research was conducted in the absence of any commercial or financial relationships that could be construed as a potential conflict of interest.

## Footnotes

1 http://jspecies.ribohost.com/jspeciesws/

2 http://ggdc.dsmz.de/distcalc2.php
