# Supplementary material for: Reclassification of Paenibacillus riograndensis as a Genomovar of Paenibacillus sonchi: Genome-Based Metrics Improve Bacterial Taxonomic Classification
Source: Front Microbiol. 2017 Oct 4;8:1849. doi: 10.3389/fmicb.2017.01849 (PMC5632714; doi:10.3389/fmicb.2017.01849)
Supplement: Supplementary file 1 [file Table_1.pdf]

Supplementary Table S1. Genomes overview.

Genome	Length	Contig number	N50	Gene number	GC content (%)	Coverage (x)	Completeness (%)*	Accession number
Paenibacillus borealis DSM 13188^T	8156808	1	8156808	6945	51.4	106	ND	NZ_CP009285
Paenibacillus durus ATCC 35681	5575484	1	5575484	5237	51	14.2	ND	NZ_CP011114
Paenibacillus durus DSM 1735^T**	6038347/15151	2	6038347	5356/26	50.8/49.1	98/107	ND	NZ_CP009288/NZ_CP009289
Paenibacillus forsythiae T98^T	5081302	896	11184	5102	52.9	115.5	ND	NZ_ASSC00000000
Paenibacillus graminis DSM 15220^T	7166454	1	7166454	5763	50.57	136	98.68	CP009287
Paenibacillus jilunlii DSM 23019^T	7076180	124	201315	6189	50.88	66	99.8	LIPY00000000
Paenibacillus odorifer DSM 15391^T	6812473	1	6812473	5945	44.2	240	ND	NZ_CP009428
Paenibacillus polymyxa ATCC 842^T	5903580	13	5894477	5206	44.93	600	99.85	NZ_AFOX00000000
Paenibacillus riograndensis SBR5^T	7919576	1	7919576	6691	50.97	198	99.22	LN831776
Paenibacillus sabinae T27^T	5270569	1	5270569	4857	52.6	263.1	ND	NZ_CP004078
Paenibacillus sonchi X19-5^T	7507281	1327	13012	7242	50.36	78.9	98.6	NZ_AJTY00000000
Paenibacillus sp. CAS34	7381498	149	138730	6377	50.83	96	99.8	LIRB00000000
Paenibacillus sp. CAR114	7349595	2107	5501	7355	50.99	10	97.61	LIRA00000000
Paenibacillus sp. HW567	6836219	1	6836219	5500	50.65	-	99.66	NZ_KB910518
Paenibacillus stellifer DSM 14472^T	5658798	1	5658798	5167	53.5	160	ND	NZ_CP009286
Paenibacillus wynnii DSM 18334^T	5988861	3	2616840	5519	44.9	150	ND	NZ_JQCR00000000
Paenibacillus zanthoxyli JH29^T	5045676	714	13835	5124	50.9	117.2	ND	NZ_ASSD00000000

* computed by Checkm. ND – not defined
** *Paenibacillus durus* DSM 1735 has a chromosome and a plasmid
