# Supplementary material for: Reclassification of Paenibacillus riograndensis as a Genomovar of Paenibacillus sonchi: Genome-Based Metrics Improve Bacterial Taxonomic Classification
Source: Front Microbiol. 2017 Oct 4;8:1849. doi: 10.3389/fmicb.2017.01849 (PMC5632714; doi:10.3389/fmicb.2017.01849)
Supplement: Supplementary file 2 [file Table_2.pdf]

Supplementary Table S2. Locus tags of *gyrB*, *recA*, *recN* and *rpoB* genes of *Paenibacillus spe*cies utilized in this study.

Strain	Locus tag			
	gyrB	*recA*	*recN*	*rpoB*
P. borealis DSM 13188^T	PBOR_00035	PBOR_21090	PBOR_24505	PBOR_32900
P. durus DSM 1735^T	PDUR_00030	PDUR_16170	PDUR_18350	PDUR_24495
P. durus ATCC 35681	VK70_22230	VK70_11695	VK70_13840	VK70_26005
P. forsythiae T98^T	L692_RS0121380	L692_RS0124190	L692_RS0124870	L692_RS0104555
P. graminis DSM 15220^T	PGRAT_00030	PGRAT_18240	PGRAT_21715	PGRAT_29390
P. jilunlii DSM 23019^T	AML91_23150	AML91_12595	AML91_27600	AML91_04045
P. odorifer DSM 15391^T	PODO_RS00030	PODO_RS17645	PODO_21510	PODO_RS27065
P. polymyxa ATCC 842^T	PPT_RS0127605	PPT_RS0110780	PPT_RS0117095	PPT_RS0124415
Paenibacillus sp. CAR114	AMQ83_15570	AMQ83_02105	AMQ83_33145	AMQ83_22310
Paenibacillus sp. CAS34	AMQ84_06735	AMQ84_24835	AMQ84_18925	AMQ84_09005
P. riograndensis SBR5	PRIO_0006	PRIO_4000	PRIO_4666	PRIO_6145
P. sabinae T27^T	PSAB_RS00030	PSAB_RS14450	PSAB_16580	PSAB_RS16600
P. sonchi X19-5^T	IC30_RS05585	IC30_RS13070	IC30_RS15600	IC30_RS02590
Paenibacillus sp. HW567	B212_RS0127585	B212_RS0109905	B212_RS0106470	B212_RS0100150
P. stellifer DSM 14472^T	PSTEL_00030	PSTEL_16060	PSTEL_18190	PSTEL_24175
P. wynnii DSM 18334^T	PWYN_11990	PWYN_22895	PWYN_25770	PWYN_15180
P. zanthoxyli JH29^T	L691_RS0116235	L691_RS0109760	L691_RS0102340	L691_RS0125345
