# Supplementary material for: Reclassification of Paenibacillus riograndensis as a Genomovar of Paenibacillus sonchi: Genome-Based Metrics Improve Bacterial Taxonomic Classification
Source: Front Microbiol. 2017 Oct 4;8:1849. doi: 10.3389/fmicb.2017.01849 (PMC5632714; doi:10.3389/fmicb.2017.01849)
Supplement: Supplementary file 3 [file Table_3.pdf]

Supplementary Table S3. GI numbers of AMPHORA proteins of *Paenibacillus* species utilized in this study.

Strain	DnaG	Frr	InfC	NusA	Pgk	PyrG	RplA	RplB
P. borealis DSM 13188[T]	754859331	754854116	754850397	754854097	754849202	754849164	746250247	746250301
P. durus DSM 1735[T]	754846549	647238903	754844344	754845795	754843233	754843181	754847243	754847235
P. durus ATCC 35681	647242829	647245047	647241055	647245666	647249440	647245647	647249630	647242002
P. forsythiae T98[T]	647248304	647253450	647246302	647248886	647252851	647248856	647240434	647247527
P. graminis DSM 15220[T]	686559797	686558699	686556791	686558691	686555675	686555627	686560546	686560528
P. jilunlii DSM 23019[T]	985071084	985076339	985074990	985076347	985075555	985080170	985079685	985079702
P. odorifer DSM 15391[T]	738792603	738797346	738788247	738797327	738795966	738788679	738789255	738789293
P. polymyxa ATCC 842[T]	518517842	504412567	494671574	515238010	515236031	515231984	515232197	515232217
Paenibacillus sp. CAR114	985090956	985085838	985086532	985092057	985085423	985090247	985088596	985085561
Paenibacillus sp. CAS34	985076507	985073232	985071026	985073224	985079618	985081862	985079504	985079487
P. riograndensis SBR5[T]	806914966	806913835	806911379	806913827	806910016	806909962	806915892	806915874
P. sabinae T27[T]	644579371	644577480	644571560	644577438	644567133	644566919	644582159	644582098
P. sonchi X19-5[T]	748295329	521103272	521096473	748294937	521094683	748290525	521096179	521096162
Paenibacillus sp. HW567	518751148	518752445	518755027	518752455	518756687	518756748	518749988	518750006
P. stellifer DSM 14472[T]	740912477	740911435	740909167	740911420	740907753	740907710	740913789	740913761
P. wynnii DSM 18334[T]	738764683	738763154	738755867	738763137	738757781	738757912	738759511	738759570
P. zanthoxyli JH29[T]	647241814	647238903	647241055	647240486	647238525	647239812	647240434	647242002

Strain	RplC	RplD	RplE	RplF	RplK	RplL	RplM	RplN
P. borealis DSM 13188[T]	754859499	754857217	754857215	738832030	521096180	746250254	754776072	518750015
P. durus DSM 1735[T]	754847237	754847236	738689889	644582054	647240435	647240432	647242021	518750015
P. durus ATCC 35681	647242000	647243508	647243505	644582054	647240435	647249633	647242021	518750015
P. forsythiae T98[T]	647247531	647247529	647243505	647247515	647240435	647247549	647242021	518750015
P. graminis DSM 15220[T]	686560531	686560530	686560519	686560517	686560547	686560544	686560501	686560521
P. jilunlii DSM 23019[T]	985079699	985079700	985079710	985079712	985079684	985079687	985079728	985079709
P. odorifer DSM 15391[T]	754768297	738789288	738789310	738789314	738789253	738789259	738789341	738789308
P. polymyxa ATCC 842[T]	503077245	503077244	494674631	503138602	494674655	503138614	503138590	494674633
Paenibacillus sp. CAR114	985085564	985085563	985088476	985088478	985088597	985088594	985091354	985088475
Paenibacillus sp. CAS34	985079490	985079489	985079479	985079477	985079505	985079502	985079462	985079480
P. riograndensis SBR5[T]	806915877	806915876	806915865	806915863	806915893	806915890	806915847	806915867
P. sabinae T27[T]	644582108	644582105	644582063	644582054	644582162	644582153	644581914	518750015
P. sonchi X19-5[T]	521096165	521096164	521096158	740811775	521096180	748290936	521096146	521096159
Paenibacillus sp. HW567	518750003	518750004	518750019	518750021	518749986	518749990	518750042	518750015
P. stellifer DSM 14472[T]	740913767	740913765	740913749	740913747	647240435	740913785	740913724	740913750
P. wynnii DSM 18334[T]	917481601	738759563	738759600	738759607	738759508	738759517	738759657	738759597
P. zanthoxyli JH29[T]	647242000	647242001	647242007	644582054	647240435	647240432	647242021	518750015

Strain	RplP	RplS	RplT	RpmA	RpoB	RpsB	RpsC	RpsE
P. borealis DSM 13188^T	746250311	746261172	738755861	521098381	754857223	738801471	746250309	738759620
P. durus DSM 1735^T	647242005	644577682	754844346	647236092	754847241	754845814	647243506	754847233
P. durus ATCC 35681	647242005	647240063	644571570	647236092	820147112	647238899	647243506	647243502
P. forsythiae T98^T	647247521	647246393	647246305	647250611	647247545	647253447	647247523	647242009
P. graminis DSM 15220^T	686560524	686558756	686556793	686559950	686560542	686558702	686560525	686560515
P. jilunlii DSM 23019^T	985079706	985076284	985074992	985078496	985079689	985076336	985079705	985079714
P. odorifer DSM 15391^T	738789304	738797448	738788242	738793994	740788177	738797351	740788168	738759620
P. polymyxa ATCC 842^T	494674636	494672057	494671576	503076671	515232203	503074989	494674637	503138600
Paenibacillus sp. CAR114	985088472	985087631	985086534	985087559	985087687	985085841	985088471	985088480
Paenibacillus sp. CAS34	985079483	985073286	985071024	985080821	985079500	985073235	985079484	985079475
P. riograndensis SBR5^T	806915870	806913894	806911381	806915150	806915888	806913838	806915871	806915861
P. sabinae T27^T	644582084	644577682	644571570	644579869	644582147	644577490	644582088	644582047
P. sonchi X19-5^T	521096161	518752371	748298160	521098381	748290938	521103275	518750009	518750024
Paenibacillus sp. HW567	518750011	518752371	518755025	518750850	655082470	518752441	518750009	518750024
P. stellifer DSM 14472^T	740913751	740911534	740909168	740912720	740913783	740911441	740913753	740913740
P. wynnii DSM 18334^T	738759592	738763429	738755861	738765013	738759525	738763159	738759581	738759620
P. zanthoxyli JH29^T	647242005	647240063	644571570	647236092	647241993	647238899	647242004	647242009

Strain	RpsI	RpsJ	RpsK	RpsM	RpsS	SmpB	Tsf
P. borealis DSM 13188^T	754776071	516288767	518750035	647258050	746250304	746248389	754854119
P. durus DSM 1735^T	647242022	493727136	647242017	647242016	644582095	647249744	738689564
P. durus ATCC 35681	647242022	493727136	647242017	647242016	644582095	647249744	647245048
P. forsythiae T98^T	647242022	493727136	644581926	647242016	644582095	647250208	647253448
P. graminis DSM 15220^T	686560500	686560532	686560505	686560506	686560527	686555681	686558701
P. jilunlii DSM 23019^T	985079729	985079698	985079724	985079723	985079703	985075549	985076337
P. odorifer DSM 15391^T	647258047	516288767	518750035	738789335	740788169	738796000	738797349
P. polymyxa ATCC 842^T	503138589	491688419	494674619	494674620	494674639	494670219	503136048
Paenibacillus sp. CAR114	985091355	985085565	985091352	985091351	985088469	AMQ83_21000*	985085840
Paenibacillus sp. CAS34	985079461	985079491	985079465	985079466	985079486	985079612	985073234
P. riograndensis SBR5^T	806915846	806915878	806915851	806915852	806915873	806910023	806913837
P. sabinae T27^T	644581911	493727136	644581926	644581929	644582095	644567166	644577486
P. sonchi X19-5^T	521096145	493727136	521096149	521096150	518750007	521096868	521103274
Paenibacillus sp. HW567	518750043	491688419	518750035	518750034	518750007	518756679	518752442
P. stellifer DSM 14472^T	740913723	493727136	647242017	647242016	740913759	740907759	740911439
P. wynnii DSM 18334^T	738759660	738759557	518750035	738759643	738759576	738757766	738763157
P. zanthoxyli JH29^T	647242022	493727136	647242017	647242016	644582095	647239629	647238901

*locus tag annotated as pseudogene, because of an internal stop codon
