# Supplementary material for: Reclassification of Paenibacillus riograndensis as a Genomovar of Paenibacillus sonchi: Genome-Based Metrics Improve Bacterial Taxonomic Classification
Source: Front Microbiol. 2017 Oct 4;8:1849. doi: 10.3389/fmicb.2017.01849 (PMC5632714; doi:10.3389/fmicb.2017.01849)
Supplement: Supplementary file 4 [file Table_4.pdf]

Supplementary Table S4. Comparison of phenotypic characteristics of *Paenibacillus* species among different reports.

	Paenibacillus sp. CAR114	*Paenibacillus* sp. CAS34	*P. riograndensis* SBR5^T			*P. sonchi* X19-5^T			*P. jilunlii* DSM 23019^T			*P. graminis* DSM 15220^T						*P. polymyxa* ATCC 842^T					
	1*	1*	1	2*	3	1	3	4*	1	3*	6	1	3	5*	8#	10	11	1	7	8	9	11	12
Acid production from:																							
D-Glucose	+	+	+	+	+	+	-	-	+	+	+	+	+	+	+	ND	+	+	+	+	+	+	ND
D-Sorbitol	+	+	+	ND	+	+	-	ND	+	-	V	+	-	-	-	-	-	+	+	+	+	+	-
D-Xylose	+	+	+	+	+	+	-	-	+	+	+	+	+	+	-	+	ND	+	+	-	-	ND	ND
Glycerol	+	+	+	+	+	+	-	ND	+	+	+	+	+	+	-	+	-	+	+	+	+	+	+
Lactose	+	+	+	+	+	+	-	-	+	+	+	+	+	+	ND	+	ND	+	+	+	+	+	ND
Maltose	+	+	+	+	+	+	-	-	+	+	+	+	+	+	+	ND	+	+	+	+	+	+	ND
Mannitol	+	+	+	+	ND	+	ND	ND	+	+	+	+	ND	+	ND	+	ND	+	+	ND	ND	ND	ND
Sucrose	+	+	+	+	+	+	-	-	+	+	+	+	-	+	-	ND	-	+	+	+	+	+	+
Catalase activity	+	+	+	ND	ND	+	ND	-	+	-	+	+	ND	+	+	ND	ND	+	ND	+	ND	ND	ND
Growth at 3% NaCl	V	V	V	ND	+	V	+	+	V	+	+	V	+	ND	ND	ND	ND	+	ND	+	ND	ND	+
Growth at 5% NaCl	-	-	-	-	ND	-	ND	-	-	-	-	-	ND	ND	ND	ND	ND	-	ND	-	ND	ND	ND
Hydrolysis of casein	+	+	+	-	-	+	+	+	+	-	V	+	-	ND	-	-	-	+	+	-	-	-	+
Hydrolysis of starch	-	+	+	+	+	-	-	+	-	+	+	+	-	+	-	+	ND	+	+	-	-	ND	+
Nitrate reduction	+	+	+	-	-	+	-	-	v	+	+	+	+	+	+	+	ND	+	+	+	+	ND	+
Voges-Proskauer test	-	-	-	-	+	-	+	+	-	-	-	-	-	ND	-	ND	-	-	+	+	+	+	+

Biochemical data obtained from this study and from literature. Asterisks indicate the original report. Underlined numbers refer to data reproduced from other studies, as detailed below. Hash symbol indicates the biochemical profile containing typographic errors. The results of biochemical tests are shown as positive "+", negative "-", variable "V", or not determined "ND". Results obtained in our study that contradicted those obtained in other reports are highlighted in gray.

1 - this study;

2. Beneduzi, A. et al. *Paenibacillus riograndensis* sp. nov., a nitrogen-fixing species isolated from the rhizosphere of *Triticum aestivum*. *Int. J. Syst. Evol. Microbiol*. **60**, 128–33 (2010);

3. Jin, H.-J., Zhou, Y.-G., Liu, H.-C. & Chen, S.-F. *Paenibacillus jilunlii* sp. nov., a nitrogen-fixing species isolated from the rhizosphere of *Begonia semperflorens*. *Int. J. Syst. Evol. Microbiol*. **61**, 1350–5 (2011);

4. Hong, Y.-Y. et al. *Paenibacillus sonchi* sp. nov., a nitrogen-fixing species isolated from the rhizosphere of *Sonchus oleraceus*. *Int. J. Syst. Evol. Microbio*l. **59**, 2656–61 (2009);

5. Berge, O., Guinebretière, M.-H., Achouak, W., Normand, P. & Heulin, T. *Paenibacillus graminis* sp. nov. and *Paenibacillus odorifer* sp. nov., isolated from plant roots, soil and food. *Int. J. Syst. Evol. Microbiol*. **52**, 607–16 (2002);

6. Kong, B. H. et al. *Paenibacillus typhae* sp. nov., isolated from roots of *Typha angustifolia* L. *Int. J. Syst. Evol. Microbiol*. **63**, 1037–44 (2013);

7. Elo, S. et al. *Paenibacillus borealis* sp. nov., a nitrogen-fixing species isolated from spruce forest humus in Finland. *Int. J. Syst. Evol. Microbiol*. **51**, 535–45 (2001);

8. Ma, Y., Xia, Z., Liu, X. & Chen, S. *Paenibacillus sabinae* sp. nov., a nitrogen-fixing species isolated from the rhizosphere soils of shrubs. *Int. J. Syst. Evol. Microbiol*. **57**, 6–11 (2007) **(data from von der Weid et al., 2002)**;

9. Ma, Y., Zhang, J. & Chen, S. *Paenibacillus zanthoxyli* sp. nov., a novel nitrogen-fixing species isolated from the rhizosphere of *Zanthoxylum simulans*. *Int. J. Syst. Evol. Microbiol*. **57**, 873–877 (2007);

10 - von der Weid, I., Duarte, G. F., van Elsas, J. D. & Seldin, L. *Paenibacillus brasilensis* sp. nov., a novel nitrogen-fixing species isolated from the maize rhizosphere in Brazil. *Int. J. Syst. Evol. Microbiol*. **52**, 2147–53 (2002) (**data from Berge et al., 2002**);

11. Ma, Y.-C. & Chen, S.-F. *Paenibacillus forsythiae* sp. nov., a nitrogen-fixing species isolated from rhizosphere soil of *Forsythia mira*. *Int. J. Syst. Evol. Microbiol.* **58**, 319–323 (2008);

12. Rodríguez-Díaz, M. et al. *Paenibacillus wynnii* sp. nov., a novel species harbouring the nifH gene, isolated from Alexander Island, Antarctica. *Int. J. Syst. Evol. Microbiol*. **55**, 2093–9 (2005).
