# Supplementary material for: Reclassification of Paenibacillus riograndensis as a Genomovar of Paenibacillus sonchi: Genome-Based Metrics Improve Bacterial Taxonomic Classification
Source: Front Microbiol. 2017 Oct 4;8:1849. doi: 10.3389/fmicb.2017.01849 (PMC5632714; doi:10.3389/fmicb.2017.01849)
Supplement: Supplementary file 5 [file Table_5.pdf]

Supplementary Table S5. Abilities related to plant growth promotion of *Paenibacillus* strains used in this study.

Bacterial strain	Phosphate solubilization	Siderophore production	IC production (μg ml^{-1})	ARA (nmol C_2H_4 mg protein)
P. riograndensis SBR5^T	negative	negative	8.91 ± 1.58	45.19 ± 4.00
P. sonchi X19-5^T	negative	negative	10.81 ± 1.91	19.08 ± 21.2
Paenibacillus sp. CAR114	positive	negative	7.02 ± 1.24	65.31 ± 4.73
Paenibacillus sp. CAS34	positive	negative	9.76 ± 1.73	81.56 ± 8.88

IC (indolic compounds); ARA (acetylene reduction assay)
