# Supplementary material for: Reclassification of Paenibacillus riograndensis as a Genomovar of Paenibacillus sonchi: Genome-Based Metrics Improve Bacterial Taxonomic Classification
Source: Front Microbiol. 2017 Oct 4;8:1849. doi: 10.3389/fmicb.2017.01849 (PMC5632714; doi:10.3389/fmicb.2017.01849)
Supplement: Supplementary file 6 [file Table_6.pdf]

Supplementary Table S6. Fatty acid composition (%) of selected members of the genus *Paenibacillus*.

	P. riograndensis SBR5[T]		*P. sonchi* X19-5[T]		*P. graminis* DSM 15220[T]		*P. jilunlli* DSM 23019[T]		*P. polymyxa* ATCC 842[T]	
Fatty acid	1	2	2	3	2	3	2	4	5	6
Saturated straight-chain										
C14:0	8.0	6.96	3.61	4.31	7.35	3.69	5.22	3.9	1.5	0.7
C16:0	17.6	16.78	8.58	19.09	7.06	14.98	11.78	9.4	6.5	9.1
Saturated iso-branched										
iso-C14:0	3.9	4.97	22.12	9.80	28.48	12.20	15.03	9.4	1.9	0.6
iso-C15:0	10.3	6.29	4.71	3.35	4.97	4.22	4.30	5.1	7.8	5.5
iso-C16:0	9.0	6.53	16.10	15.85	11.18	13.42	13.32	10.2	11.3	7.7
Saturated anteiso-branched										
anteiso-C15:0	45.7	44.45	35.95	39.36	36.23	42.00	30.92	54.4	55.2	49.9
anteiso-C17:0	3.16	1.79	1.07	2.01	0.37	1.45	1.88	2.2	8.8	16.7

1. Beneduzi, A. et al. *Paenibacillus riograndensis* sp. nov., a nitrogen-fixing species isolated from the rhizosphere of *Triticum aestivum*. *Int. J. Syst. Evol. Microbiol*. **60**, 128–33 (2010);

2. Jin, H.-J., Zhou, Y.-G., Liu, H.-C. & Chen, S.-F. *Paenibacillus jilunlii* sp. nov., a nitrogen-fixing species isolated from the rhizosphere of *Begonia semperflorens*. *Int. J. Syst. Evol. Microbiol*. **61**, 1350–5 (2011);

3. Hong, Y.-Y. et al. *Paenibacillus sonchi* sp. nov., a nitrogen-fixing species isolated from the rhizosphere of *Sonchus oleraceus*. *Int. J. Syst. Evol. Microbiol*. **59**, 2656–61 (2009);

4. Kong, B. H. et al. *Paenibacillus typhae* sp. nov., isolated from roots of *Typha angustifolia* L. *Int. J. Syst. Evol. Microbiol.* **63**, 1037–44 (2013);

5. Elo, S. et al. *Paenibacillus borealis* sp. nov., a nitrogen-fixing species isolated from spruce forest humus in Finland. *Int. J. Syst. Evol. Microbiol.* **51**, 535–45 (2001);

6. Lee, M. et al. *Paenibacillus ginsengisoli* sp. nov., a novel bacterium isolated from soil of a ginseng field in Pocheon Province, South Korea. *Antonie Van Leeuwenhoek* **91**, 127–35 (2007).
