# Supplementary material for: Reclassification of Paenibacillus riograndensis as a Genomovar of Paenibacillus sonchi: Genome-Based Metrics Improve Bacterial Taxonomic Classification
Source: Front Microbiol. 2017 Oct 4;8:1849. doi: 10.3389/fmicb.2017.01849 (PMC5632714; doi:10.3389/fmicb.2017.01849)
Supplement: Supplementary file 7 [file Table_7.pdf]

Supplementary Table S7. Pairwise comparisons of 16S rRNA genes of selected members of *Paenibacillus* species.

	P. riograndensis SBR5^T	*P. sonchi* X19-5^T	*Paenibacillus* sp. CAR114	*Paenibacillus* sp. CAS34	*P. graminis* DSM 15220^T	*P. jilunlii* DSM 23019^T
P. sonchi X19-5^T	**0.99**					
Paenibacillus sp. CAR114	**0.992**	**0.994**				
Paenibacillus sp. CAS34	**0.993**	**0.996**	**0.997**			
P. graminis DSM 15220^T	**0.99**	**0.991**	**0.994**	**0.995**		
P. jilunlii DSM 23019^T	**0.985**	**0.992**	**0.993**	**0.99**	**0.992**	
P. polymyxa ATCC 842^T	0.942	0.948	0.945	0.947	0.945	0.945

Identity values ≥ 0.985, the threshold for species demarcation, are highlighted
