# Supplementary material for: Reclassification of Paenibacillus riograndensis as a Genomovar of Paenibacillus sonchi: Genome-Based Metrics Improve Bacterial Taxonomic Classification
Source: Front Microbiol. 2017 Oct 4;8:1849. doi: 10.3389/fmicb.2017.01849 (PMC5632714; doi:10.3389/fmicb.2017.01849)
Supplement: Supplementary file 8 [file Table_8.pdf]

Supplementary Table S8. ANI values based on blast alignments of *Paenibacillus* genomes.

	P. riograndensis SBR5^T	*P. sonchi* X19-5^T	*Paenibacillus* sp. CAR114	*Paenibacillus* sp. CAS34	*P. graminis* DSM 15220^T	*P. jilunlii* ATCC 23019^T	*P. polymyxa* ATCC 842^T	*Paenibacillus* sp. HW567
P. riograndensis SBR5^T	*	**96.09 [70.37]**	**96.31 [71.47]**	**97.01 [80.40]**	91.42 [69.39]	92.43 [71.74]	68.84 [24.52]	80.94 [53.95]
P. sonchi X19-5^T	**96.28 [77.67]**	*	**95.61 [70.63]**	**96.32 [78.48]**	91.70 [72.48]	92.99 [75.94]	68.84 [24.94]	81.01 [54.58]
Paenibacillus sp. CAR114	**96.12 [83.71]**	**95.35 [74.31]**	*	**97.70 [91.20]**	90.82 [72.85]	91.85 [75.61]	68.69 [24.83]	80.52 [55.04]
Paenibacillus sp. CAS34	**97.27 [84.96]**	**96.42 [75.53]**	**98.21 [82.02]**	*	91.75 [73.73]	92.80 [76.46]	68.73 [25.27]	80.98 [55.45]
P. graminis DSM 15220^T	91.76 [75.71]	92.06 [71.01]	91.38 [67.70]	91.84 [76.03]	*	93.02 [78.67]	69.03 [26.79]	81.19 [56.38]
P. jilunlii ATCC 23019^T	92.76 [79.29]	93.31 [75.58]	92.45 [70.39]	92.91 [79.08]	92.96 [79.55]	*	68.86 [26.56]	81.26 [57.45]
P. polymyxa ATCC 842^T	68.57 [31.36]	68.88 [29.01]	68.61 [27.69]	68.63 [30.88]	68.56 [31.49]	68.79 [30.80]	*	68.44 [29.60]
Paenibacillus sp. HW567	81.24 [60.74]	81.28 [55.45]	80.89 [52.99]	81.18 [59.10]	81.22 [58.35]	81.19 [59.25]	68.61 [25.75]	*

ANIb values ≥ 95%, the threshold for species demarcation, are highlighted. Aligned percentage are in brackets.
