# Supplementary material for: Reclassification of Paenibacillus riograndensis as a Genomovar of Paenibacillus sonchi: Genome-Based Metrics Improve Bacterial Taxonomic Classification
Source: Front Microbiol. 2017 Oct 4;8:1849. doi: 10.3389/fmicb.2017.01849 (PMC5632714; doi:10.3389/fmicb.2017.01849)
Supplement: Supplementary file 9 [file Table_9.pdf]

Supplementary Table S9. MiSI values based on *Paenibacillus* ortholog genes.

	P. riograndensis SBR5^T	*P. sonchi* X19-5^T	*Paenibacillus* sp. CAR114	*Paenibacillus* sp. CAS34	*P. graminis* DSM 15220^T	*P. jilunlii* ATCC 23019^T	*P. polymyxa* ATCC 842^T	*Paenibacillus* sp. HW567
P. riograndensis SBR5^T		**97.29 [0.71]**	**97.45 [0.61]**	**97.96 [0.79]**	93.04 [0.68]	93.94 [0.73]	71.9 [0.21]	83 [0.56]
P. sonchi X19-5^T	**97.31 [0.78]**		**96.78 [0.6]**	**97.29 [0.76]**	93.34 [0.69]	94.33 [0.75]	72.1 [0.22]	83.09 [0.55]
Paenibacillus sp. CAR114	**97.48 [0.83]**	**96.78 [0.75]**		**98.73 [0.86]**	92.74 [0.71]	93.65 [0.75]	72.13 [0.22]	82.88 [0.57]
Paenibacillus sp. CAS34	**97.98 [0.9]**	**97.28 [0.8]**	**98.73 [0.72]**		93.07 [0.78]	93.98 [0.83]	71.82 [0.24]	83.03 [0.62]
P. graminis DSM 15220^T	93.04 [0.85]	93.34 [0.79]	92.74 [0.65]	93.07 [0.85]		94.08 [0.86]	71.9 [0.26]	83.11 [0.65]
P. jilunlii ATCC 23019^T	93.94 [0.87]	94.33 [0.82]	93.66 [0.66]	93.98 [0.86]	94.08 [0.83]		72.11 [0.25]	83.12 [0.64]
P. polymyxa ATCC 842^T	71.91 [0.29]	72.11 [0.28]	72.13 [0.22]	71.82 [0.29]	71.9 [0.29]	72.1 [0.29]		71.67 [0.27]
Paenibacillus sp. HW567	82.99 [0.71]	83.09 [0.63]	82.88 [0.52]	83.02 [0.68]	83.11 [0.65]	83.11 [0.67]	71.68 [0.24]	

MiSI values ≥ 96.5%, the threshold for species demarcation, are highlighted. Aligned fraction are in brackets.
