# Supplementary material for: Reclassification of Paenibacillus riograndensis as a Genomovar of Paenibacillus sonchi: Genome-Based Metrics Improve Bacterial Taxonomic Classification
Source: Front Microbiol. 2017 Oct 4;8:1849. doi: 10.3389/fmicb.2017.01849 (PMC5632714; doi:10.3389/fmicb.2017.01849)
Supplement: Supplementary file 10 [file Table_10.pdf]

Supplementary Table S10. dDDH values among *Paenibacillus* genomes.

	P. riograndensis SBR5^T	*P. sonchi* X19-5^T	*Paenibacillus* sp. CAR114	*Paenibacillus* sp. CAS34	*P. graminis* DSM 15220^T	*P. jilunlii* ATCC 23019^T	*P. polymyxa* ATCC 842^T
P. sonchi X19-5^T	**73.2 [2.92]**						
Paenibacillus sp. CAR114	**72.6 [2.92]**	68.2 [2.92]					
Paenibacillus sp. CAS34	**78.1 [2.84]**	**72.8 [2.92]**	**84.3 [2.58]**				
P. graminis DSM 15220^T	47.6 [2.59]	51.6 [2.66]	45.8 [2.57]	47.4 [2.59]			
P. jilunlii ATCC 23019^T	51.3 [2.65]	53.1 [2.69]	49.1 [2.62]	51.1 [2.65]	51.6 [2.66]		
P. polymyxa ATCC 842^T	23.5 [2.38]	22.1 [2.36]	22.3 [2.36]	21.9 [2.35]	24.4 [2.39]	22.8 [2.37]	
Paenibacillus sp. HW567	26.4 [2.42]	26.9 [2.42]	26.5 [2.42]	26.2 [2.41]	26.5 [2.42]	26.4 [2.42]	21.9 [2.35]

dDDH values ≥ 70%, the threshold for species demarcation, are highlighted. Interval of confidence values are in brackets.
