# Supplementary material for: Reclassification of Paenibacillus riograndensis as a Genomovar of Paenibacillus sonchi: Genome-Based Metrics Improve Bacterial Taxonomic Classification
Source: Front Microbiol. 2017 Oct 4;8:1849. doi: 10.3389/fmicb.2017.01849 (PMC5632714; doi:10.3389/fmicb.2017.01849)
Supplement: Supplementary file 11 [file Table_11.pdf]

Supplementary Table S11. ANI and 16S rRNA identity values of pairwise comparisons of *Paenibacillus* species.

Taxon 1	Taxon 2	ANI (%)	16S rRNA gene identity (%)
P. riograndensis SBR5^{T}	*P. riograndensis* CAS34	97.01	99.3
P. riograndensis SBR5^{T}	*P. riograndensis* CAR114	96.31	99.2
P. riograndensis CAS34	*P. riograndensis* CAR114	98.21	99.7
P. riograndensis SBR5^{T}	*P. sonchi* X19-5^{T}	96.09	99
P. riograndensis CAS34	*P. sonchi* X19-5^{T}	96.42	99.6
P. riograndensis CAR114	*P. sonchi* X19-5^{T}	95.35	99.4
P. riograndensis SBR5^{T}	*P. graminis* DSM 15220^{T}	91.42	99
P. riograndensis CAS34	*P. graminis* DSM 15220^{T}	91.75	99.5
P. riograndensis CAR114	*P. graminis* DSM 15220^{T}	90.82	99.4
P. sonchi X19-5^{T}	*P. graminis* DSM 15220^{T}	91.7	99.1
P. riograndensis SBR5^{T}	*P. jilunlii* DSM 23019^{T}	92.43	98.5
P. riograndensis CAS34	*P. jilunlii* DSM 23019^{T}	92.8	99
P. riograndensis CAR114	*P. jilunlii* DSM 23019^{T}	91.86	99.3
P. sonchi X19-5^{T}	*P. jilunlii* DSM 23019^{T}	93	99.2
P. graminis DSM 15220^{T}	*P. jilunlii* DSM 23019^{T}	93.02	99.2
P. riograndensis SBR5^{T}	*Paenibacillus* sp. HW567	80.94	98.5
P. riograndensis CAS34	*Paenibacillus* sp. HW567	80.98	99
P. riograndensis CAR114	*Paenibacillus* sp. HW567	80.52	98.8
P. sonchi X19-5^{T}	*Paenibacillus* sp. HW567	81.01	98.7
P. graminis DSM 15220^{T}	*Paenibacillus* sp. HW567	81.19	99.1
P. jilunlii DSM 23019^{T}	*Paenibacillus* sp. HW567	81.26	98.7
P. riograndensis SBR5^{T}	*P. borealis* DSM 13188^{T}	77.55	96.5
P. riograndensis CAS34	*P. borealis* DSM 13188^{T}	77.67	96.9
P. riograndensis CAR114	*P. borealis* DSM 13188^{T}	77.3	96.9
P. sonchi X19-5^{T}	*P. borealis* DSM 13188^{T}	77.87	96.6
P. graminis DSM 15220^{T}	*P. borealis* DSM 13188^{T}	77.92	97.1
P. jilunlii DSM 23019^{T}	*P. borealis* DSM 13188^{T}	77.83	97
Paenibacillus sp. HW567	*P. borealis* DSM 13188^{T}	77.59	97.1
P. riograndensis SBR5^{T}	*P. odorifer* DSM 15391^{T}	74.03	96.7
P. riograndensis CAS34	*P. odorifer* DSM 15391^{T}	73.93	97
P. riograndensis CAR114	*P. odorifer* DSM 15391^{T}	73.72	96.8
P. sonchi X19-5^{T}	*P. odorifer* DSM 15391^{T}	74.03	97
P. graminis DSM 15220^{T}	*P. odorifer* DSM 15391^{T}	74.24	97.2
P. jilunlii DSM 23019^{T}	*P. odorifer* DSM 15391^{T}	74.15	97.1
Paenibacillus sp. HW567	*P. odorifer* DSM 15391^{T}	74.07	97.2
P. borealis DSM 13188^{T}	*P. odorifer* DSM 15391^{T}	73.93	98.7
P. riograndensis SBR5^{T}	*P. wynnii* DSM 18334^{T}	73.36	95.1
P. riograndensis CAS34	*P. wynnii* DSM 18334^{T}	73.13	95.3
P. riograndensis CAR114	*P. wynnii* DSM 18334^{T}	73.01	95.3
P. sonchi X19-5^{T}	*P. wynnii* DSM 18334^{T}	73.32	95
P. graminis DSM 15220^{T}	*P. wynnii* DSM 18334^{T}	73.56	95.6
P. jilunlii DSM 23019^{T}	*P. wynnii* DSM 18334^{T}	73.47	95.3
Paenibacillus sp. HW567	*P. wynnii* DSM 18334^{T}	73.53	95.6
P. borealis DSM 13188^{T}	*P. wynnii* DSM 18334^{T}	73.24	96.7
P. odorifer DSM 15391^{T}	*P. wynnii* DSM 18334^{T}	74.1	96.7
P. riograndensis SBR5^{T}	*P. stellifer* DSM 14472^{T}	72.83	95.8
P. riograndensis CAS34	*P. stellifer* DSM 14472^{T}	72.75	96.1
P. riograndensis CAR114	*P. stellifer* DSM 14472^{T}	72.63	96
P. sonchi X19-5^{T}	*P. stellifer* DSM 14472^{T}	72.8	95.7
P. graminis DSM 15220^{T}	*P. stellifer* DSM 14472^{T}	72.82	96.3
P. jilunlii DSM 23019^{T}	*P. stellifer* DSM 14472^{T}	72.7	96.1
Paenibacillus sp. HW567	*P. stellifer* DSM 14472^{T}	72.69	96.5
P. borealis DSM 13188^{T}	*P. stellifer* DSM 14472^{T}	72.33	97.5

P. odorifer DSM 15391^{T}	*P. stellifer* DSM 14472^{T}	70.71	97.4
P. wynnii DSM 18334^{T}	*P. stellifer* DSM 14472^{T}	71.27	95.8
P. riograndensis SBR5^{T}	*P. durus* DSM 1735^{T}	73.23	96.2
P. riograndensis CAS34	*P. durus* DSM 1735^{T}	73.21	96.6
P. riograndensis CAR114	*P. durus* DSM 1735^{T}	72.82	96.4
P. sonchi X19-5^{T}	*P. durus* DSM 1735^{T}	73.1	96.2
P. graminis DSM 15220^{T}	*P. durus* DSM 1735^{T}	73.33	96.2
P. jilunlii DSM 23019^{T}	*P. durus* DSM 1735^{T}	73.24	95.9
Paenibacillus sp. HW567	*P. durus* DSM 1735^{T}	73.2	96.5
P. borealis DSM 13188^{T}	*P. durus* DSM 1735^{T}	72.89	97.5
P. odorifer DSM 15391^{T}	*P. durus* DSM 1735^{T}	71.98	97.5
P. wynnii DSM 18334^{T}	*P. durus* DSM 1735^{T}	72.32	95.3
P. stellifer DSM 14472^{T}	*P. durus* DSM 1735^{T}	76.39	97.5
P. riograndensis SBR5^{T}	*P. forsythiae* T98^{T}	73.27	96.7
P. riograndensis CAS34	*P. forsythiae* T98^{T}	73.33	97.1
P. riograndensis CAR114	*P. forsythiae* T98^{T}	73.13	96.8
P. sonchi X19-5^{T}	*P. forsythiae* T98^{T}	73.25	97.2
P. graminis DSM 15220^{T}	*P. forsythiae* T98^{T}	73.42	96.7
P. jilunlii DSM 23019^{T}	*P. forsythiae* T98^{T}	73.43	96.8
Paenibacillus sp. HW567	*P. forsythiae* T98^{T}	73.37	97
P. borealis DSM 13188^{T}	*P. forsythiae* T98^{T}	73	96.6
P. odorifer DSM 15391^{T}	*P. forsythiae* T98^{T}	71.79	97.1
P. wynnii DSM 18334^{T}	*P. forsythiae* T98^{T}	72	95.3
P. stellifer DSM 14472^{T}	*P. forsythiae* T98^{T}	76.78	96.8
P. durus DSM 1735^{T}	*P. forsythiae* T98^{T}	86.4	98.1
P. riograndensis SBR5^{T}	*P. sabinae* T27^{T}	73.46	96.2
P. riograndensis CAS34	*P. sabinae* T27^{T}	73.26	96.7
P. riograndensis CAR114	*P. sabinae* T27^{T}	73.05	96.6
P. sonchi X19-5^{T}	*P. sabinae* T27^{T}	73.32	96.9
P. graminis DSM 15220^{T}	*P. sabinae* T27^{T}	73.46	96.4
P. jilunlii DSM 23019^{T}	*P. sabinae* T27^{T}	73.45	96.6
Paenibacillus sp. HW567	*P. sabinae* T27^{T}	73.29	96.6
P. borealis DSM 13188^{T}	*P. sabinae* T27^{T}	73.01	96.8
P. odorifer DSM 15391^{T}	*P. sabinae* T27^{T}	71.71	97.1
P. wynnii DSM 18334^{T}	*P. sabinae* T27^{T}	72.13	95.3
P. stellifer DSM 14472^{T}	*P. sabinae* T27^{T}	76.7	97.4
P. durus DSM 1735^{T}	*P. sabinae* T27^{T}	83.64	98
P. forsythiae T98^{T}	*P. sabinae* T27^{T}	82.74	98.6
P. riograndensis SBR5^{T}	*P. zanthoxyli* JH29^{T}	73.63	96.1
P. riograndensis CAS34	*P. zanthoxyli* JH29^{T}	73.5	96.6
P. riograndensis CAR114	*P. zanthoxyli* JH29^{T}	73.21	96.5
P. sonchi X19-5^{T}	*P. zanthoxyli* JH29^{T}	73.47	96.7
P. graminis DSM 15220^{T}	*P. zanthoxyli* JH29^{T}	73.68	96.2
P. jilunlii DSM 23019^{T}	*P. zanthoxyli* JH29^{T}	73.44	96.3
Paenibacillus sp. HW567	*P. zanthoxyli* JH29^{T}	73.47	96.2
P. borealis DSM 13188^{T}	*P. zanthoxyli* JH29^{T}	72.98	96.2
P. odorifer DSM 15391^{T}	*P. zanthoxyli* JH29^{T}	72.12	96.8
P. wynnii DSM 18334^{T}	*P. zanthoxyli* JH29^{T}	72.47	95.2
P. stellifer DSM 14472^{T}	*P. zanthoxyli* JH29^{T}	76.46	96.8
P. durus DSM 1735^{T}	*P. zanthoxyli* JH29^{T}	91.44	97.6
P. forsythiae T98^{T}	*P. zanthoxyli* JH29^{T}	86.34	98.4
P. sabinae T27^{T}	*P. zanthoxyli* JH29^{T}	83.45	98.6
P. riograndensis SBR5^{T}	*P. polymyxa* ATCC 842^{T}	68.84	94.4
P. riograndensis CAS34	*P. polymyxa* ATCC 842^{T}	68.73	94.9
P. riograndensis CAR114	*P. polymyxa* ATCC 842^{T}	68.69	94.7
P. sonchi X19-5^{T}	*P. polymyxa* ATCC 842^{T}	68.84	95.1

P. graminis DSM 15220^{T}	*P. polymyxa* ATCC 842^{T}	69.03	94.7
P. jilunlii DSM 23019^{T}	*P. polymyxa* ATCC 842^{T}	68.86	94.7
Paenibacillus sp. HW567	*P. polymyxa* ATCC 842^{T}	68.61	94.7
P. borealis DSM 13188^{T}	*P. polymyxa* ATCC 842^{T}	68.73	95
P. odorifer DSM 15391^{T}	*P. polymyxa* ATCC 842^{T}	68.95	95.5
P. wynnii DSM 18334^{T}	*P. polymyxa* ATCC 842^{T}	68.87	94.6
P. stellifer DSM 14472^{T}	*P. polymyxa* ATCC 842^{T}	68.82	94.6
P. durus DSM 1735^{T}	*P. polymyxa* ATCC 842^{T}	68.8	95.7
P. forsythiae T98^{T}	*P. polymyxa* ATCC 842^{T}	68.31	96.1
P. sabinae T27^{T}	*P. polymyxa* ATCC 842^{T}	68.99	96.2
P. zanthoxyli JH29^{T}	*P. polymyxa* ATCC 842^{T}	68.91	95.6
