# Supplementary material for: Reclassification of Paenibacillus riograndensis as a Genomovar of Paenibacillus sonchi: Genome-Based Metrics Improve Bacterial Taxonomic Classification
Source: Front Microbiol. 2017 Oct 4;8:1849. doi: 10.3389/fmicb.2017.01849 (PMC5632714; doi:10.3389/fmicb.2017.01849)
Supplement: Supplementary file 12 [file Image_1.pdf]

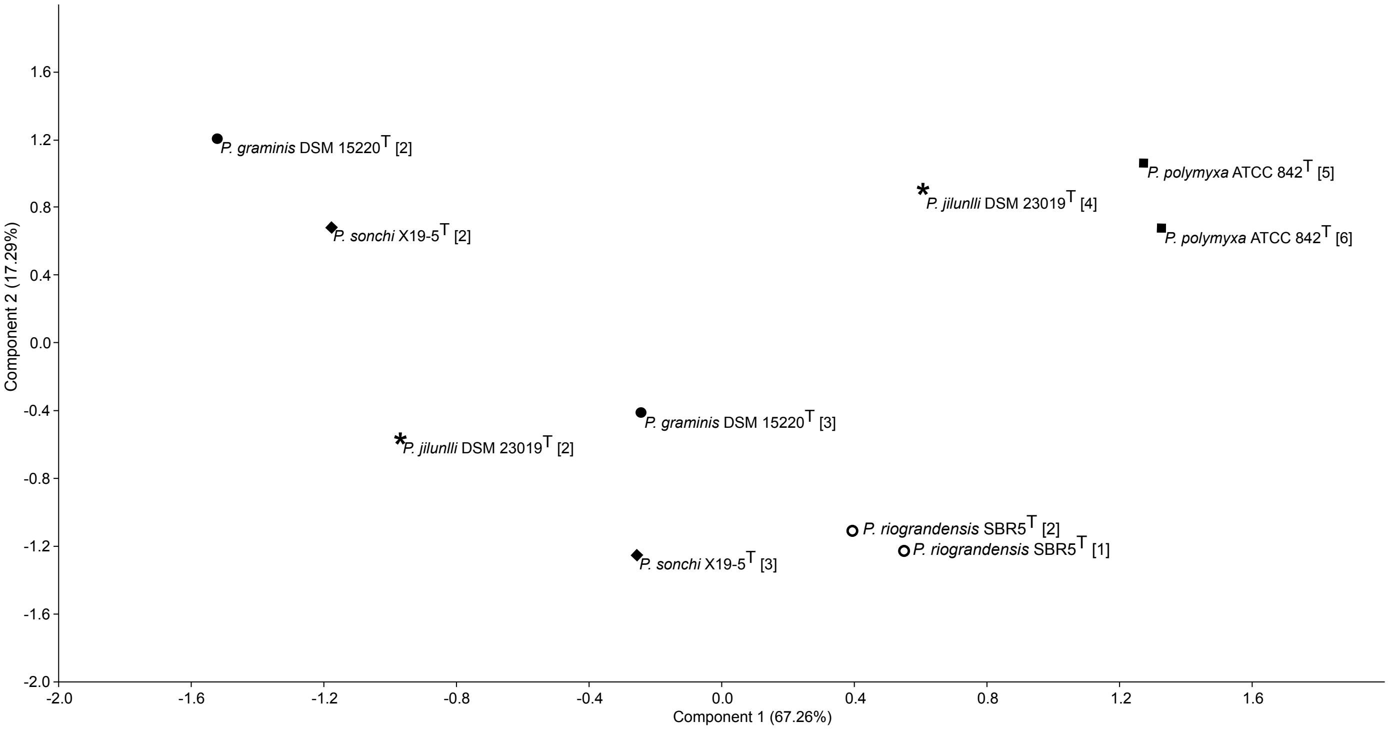

Supplementary Figure S1. Principal component analysis (PCA) derived from the fatty acid compositions of five *Paenibacillus* type-strains.

PCA verified the statistical variance-covariance among fatty acid contents and *Paenibacillus* type-strains, according to Supplementary Table S4. The following fatty acids were assessed: saturated straight-chain (C14:0, C16:0), saturated iso-branched (iso-C14:0, iso-C15:0, iso-C16:0), saturated anteiso-branched (anteiso-C15:0, anteiso-C17:0). Principal components 1 and 2 accounted for 67.26% and 17.29% of the total variation, respectively. The eigenvalues 1 and 2 were 144.005 and 37.0177.50, respectively, and the Jolliffe cut-off was 21.41.
