# Supplementary material for: Reclassification of Paenibacillus riograndensis as a Genomovar of Paenibacillus sonchi: Genome-Based Metrics Improve Bacterial Taxonomic Classification
Source: Front Microbiol. 2017 Oct 4;8:1849. doi: 10.3389/fmicb.2017.01849 (PMC5632714; doi:10.3389/fmicb.2017.01849)
Supplement: Supplementary file 13 [file Image_2.pdf]

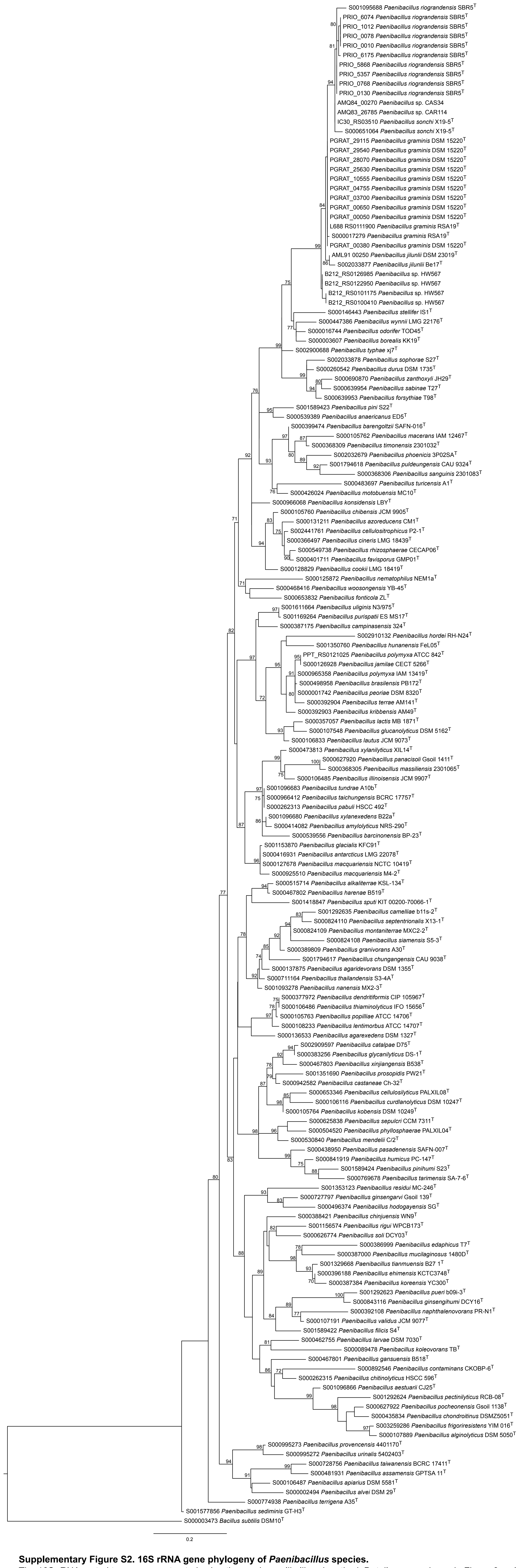

Supplementary Figure S2. 16S rRNA gene phylogeny of *Paenibacillus* species.
The 16S rRNA rooted tree was constructed using the maximum-likelihood method. Details are as shown in Figure 3, unless specified otherwise. *Bacillus subtilis* is the outgroup. An alignment containing 775 positions was utilized in this phylogenetic reconstruction.
